# Flow Regulation Performance Analysis of Microfluidic Passive Valve for High Throughput Liquid Delivery

**DOI:** 10.3390/mi13050687

**Published:** 2022-04-28

**Authors:** Qi Su, Weiran Chen, Weiping Chen, Zhijiang Jin, Zhenhao Lin

**Affiliations:** 1 State Key Laboratory of Fluid Power and Mechatronic Systems, Zhejiang University, Hangzhou 310027, China; suqi@zju.edu.cn; 2 Institute of Process Equipment, College of Energy Engineering, Zhejiang University, Hangzhou 310027, China; 3190100978@zju.edu.cn (W.C.); jzj@zju.edu.cn (Z.J.); 3 Hangzhou Worldwides Valve Co., Ltd., Huzhou 311122, China; chuang_liu@zju.edu.cn; 4 Institute of Wenzhou, Zhejiang University, Wenzhou 325036, China

**Keywords:** microfluidic passive valve (MPV), flow rate, flow characteristics, threshold pressure

## Abstract

A microfluidic passive valve (MPV) is important for precise flow control, and it determines the reliability of the microfluidic system. In this paper, a novel MPV capable of delivering a constant flow rate independently of inlet pressure changes is proposed. The flow rate of the MPV is adjusted by the difference between the fluid force on the upper surface of the valve core and the spring force. The constant flow rate of the MPV is maintained by automatically changing the size of the gap channel formed by the groove on the valve core and the baffle on the valve body. The nearly constant flow rate of the MPV is 6.26 mL/min, with a variation of 6.5% under the inlet pressure varied from 1.25 kPa to 3.5 kPa. In addition, the flow characteristics of the MPV are analyzed by numerical simulation. With the increase in the inlet pressure, the maximum velocity gradually increases, while the increment of the maximum velocity decreases. In the movement process of the valve core, the region of pressure drop becomes larger. This work has a certain reference value for the design and research of the MPVs with high throughput liquid delivery.

## 1. Introduction

Microfluidics is a new technology that uses microchannels of tens to hundreds of microns to deal with small volumes of fluids [1,2,3]. Microfluidics systems have obvious advantages, such as low cost, small space, high precision and effective flow control. It is widely used in various fields, including medicine [4], chemistry [5], and biomedicine [6], etc. In recent years, with the vigorous development of microfluidic technology, microfluidic valves have received extensive attention due to their precise control of microfluidics and low-cost fabrication, such as point-of-care tests (POCT) of tumor marker proteins [7], unidirectional transport of nanofluids [8,9,10], sweat collection microdevice for sweat colorimetric (AST) [11], rapid and accurate antibiotic-susceptibility testing [12], etc. A compact and efficient MPV is crucial for the precise manipulation of microfluidics, which directly determines the reliability of the detection results of microfluidic chips.

According to the structure and principle of fluid regulation, microfluidic valves can be divided into two categories: microfluidic active valves (MAV) and microfluidic passive valves (MPV) [13]. The MAV can change the flow resistance through an external excitation component to achieve the purpose of regulating the microfluidics, such as pneumatically actuated MAV, electrostatically actuated MAV, piezoelectric actuated MAV, magnetically actuated MAV, and so on. A pneumatically actuated MAV requiring a vacuum pump and a pneumatic actuator was proposed [14,15,16]. The vacuum pump provides the pressure source, and the pneumatic actuator drives the membrane to bend, which causes the valve to close. An electrostatically actuated MAV is mainly composed of a valve closing electrode, an opening valve electrode, and a flexible membrane [17,18,19]. The valve opens and closes by controlling the voltage applied to the membrane. A piezoelectric actuated MAV produces a large bending force and small displacement of the membrane through the piezoelectric actuator. They are mainly composed of piezoelectric actuators, membranes, and valve seats [20,21,22]. A magnetically actuated MAV consists of a permanent magnet and a flexible elastic membrane with a soft magnetic material. The membrane is deflected by magnetic force to control the opening and closing of the valve [23,24,25].

Compared to the MAV, MPV avoids the introduction of external components for microfluidic control. Therefore, the MPV is compact, reliable, maintenance-free, which requires no energy. The flow regulators of MPV are dominated by the mechanical regulators having moving components (membrane, piston, cantilever, leaflet, and so on) [26]. For the MPV with a membrane as the mechanical regulator, the internal fluid pressure drives the elastic membrane to deform, thereby controlling the flow or the switch of opening and closing. The main materials of the membrane are shape memory alloys [27,28], glass [29,30] and polydimethylsiloxane (PDMS) [31,32]. For the MPV with a piston as the mechanical regulator, the valve uses fluid pressure to move the piston with a threaded groove for microfluidic control [33]. For the MPV with a cantilever as the mechanical regulator, the valve consists of a thin cantilever and stiff stopper that form flow constriction in a microchannel [34,35,36]. The MPV with two leaflets as the mechanical regulator is called the heart valve in the medical field. The two leaflets rotate on a fixed axis under the impact of blood to realize the opening of the valve [37,38].

By a change of fluidic pathway dimensions, the flow rate of the MPV can be automatically adjusted with different pressure conditions. It has become a research hotspot in this field. Kartalov et al. [39] invented the PDMS push-up microfluidic valve, including a detour control channel, a membrane, and a fluidic channel. Yang et al. [40] designed a check microfluidic valve with a compliant flap and a rigid stopper. Doh et al. [41] proposed a parallel membrane microfluidic valve comprising two control channels and experimentally studied the variation of the flow rate with different pressures. Chappel et al. [33] presented a microfluidic passive flow control valve dedicated to the hydrocephalus treatment. It comprises a hollow cylinder, a piston engraved with a helical groove, a spring, and two fluidic connectors. Zhang et al. [42] fabricated a microfluidic passive valve with a liquid chamber, an elastic membrane, and an ellipsoidal control chamber. The elastic membrane has two orifices. Later, Lin et al. [43] optimized the structure of this valve, and studied the effect of different numbers of holes on constant flow. The purpose of the above research is to realize that the outlet flow rate remains constant when the inlet pressure reaches a certain threshold. The flow regulation capabilities of different MPV structures are shown in Table 1.

In this paper, a novel MPV capable of delivering a constant flow rate independently of inlet pressure changes is proposed. To investigate the flow characteristics of the MPV, computational fluid dynamics (CFD) is adopted to study the flow field and flow rate with the varied set of inlet pressures. This work has a certain reference value for the design and research of the MPVs with high throughput liquid delivery.

## 2. Valve Design and Numerical Methods

### 2.1. Valve Design

**Figure 1:**
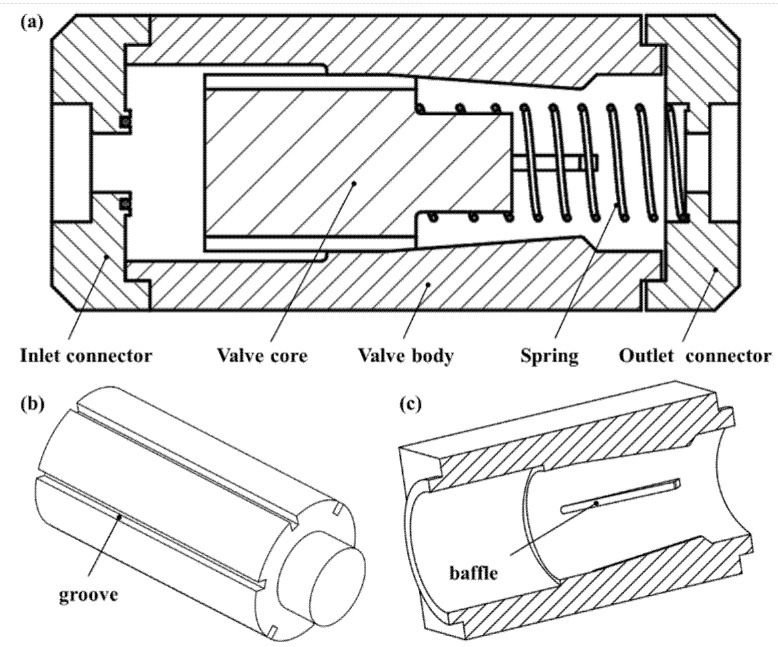
Structure of MPV: (**a**) MPV diagram, (**b**) valve core, (**c**) valve body.

The MPV main consists of an inlet connector, outlet connector, valve body, and spring and valve core, as shown in Figure 1a. The valve core is a piston-type structure. The outer wall of the valve core has several grooves with the same structure along the circumferential direction, as shown in Figure 1b. The inner wall of the valve body has several baffles with the same structure along the circumferential direction to guide the movement of the valve core. The number of baffles and the number of grooves is the same. The height of the baffle protrusion increases gradually along the flow direction of the fluid, as shown in Figure 1c. Each groove can accommodate a baffle, and a gap channel can be formed between them for the passage of fluid. The size of the gap channel can be adjusted by the axial movement of the valve core, and the gap channel can be closed when the groove is in contact with the end of the baffle. The MPV is closed by the preload force of the spring with no fluid in the valve. When the force of the fluid pressure acting on the top surface of the valve core is greater than the spring force and frictional force, the valve core moves and the valve is opened. The valve body has an inner storage cavity on the inlet side in order to store a volume of fluid around the upper part of the valve core that is submitted to pressure substantially equal to the inlet pressure. The valve body also has an outer storage cavity on the outlet side, and its function is the same as the inner storage cavity.

**Figure 2:**
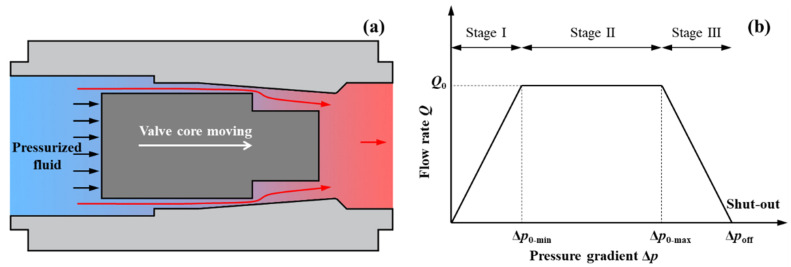
Working principle of the MPV: (**a**) Schematic valve actuation under pressurized fluid; (**b**) Ideal flow rate versus pressure gradient characteristic.

The main pressure drop inside the valve occurs when the fluid is forced to flow inside the gap channel between the groove and baffle. As presented in Figure 2a, when the inlet pressure is increased to move the valve core, the gap channel between the groove and baffle becomes smaller. Thus, the flow resistance of the valve increases. At a given temperature, the flow rate *Q* of the valve is calculated by

(1) $$Q=\frac{\Delta p}{R_{h}}=\frac{\Delta p+\delta p}{R_{h}+\delta R_{h}}$$

where △*p* = *P*_out_ − *P*_in_ refers to the pressure drop between the inlet and the outlet of MPV, *R*_h_ refers to the flow resistance of MPV. It can be seen from Equation (1) that, when the pressure gradient *δp* (i.e., inlet pressure) increases, the flow resistance *δR_h_* increases. At low pressure, the MPV has a constant and low flow resistance, as shown in stage-Ι of Figure 2b. When the pressure drop is beyond a threshold pressure △*p*_0_, the flow resistance *δR_h_* increment automatically compensates for the pressure drop increment *δp*, and the MPV can output a consistent flow rate *Q*_0_, as shown in stage-Ⅱ of Figure 2b. However, when the pressure gradient is too high, the groove is in contact with the end of the baffle and the gap channel is closed. Simultaneously, the flow resistance tends to be infinite, and the flow rate is close to zero, as shown in stage-Ⅲ of Figure 2b.

**Figure 3:**
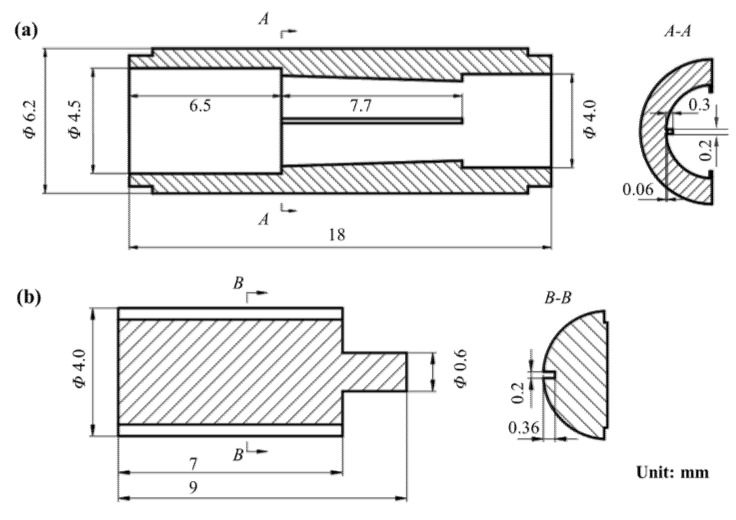
Main structure parameters of the MPV: (**a**) valve body; (**b**) valve core.

Based on the working principle of the MPV, the structure is designed in detail. The outside diameter of the valve body is 6.2 mm and the total length is 18 mm. The valve body has four sloping baffles with an inlet end height of 0.06 mm, an outlet end height of 0.3 mm, a thickness of 0.2 mm, and a total length of 7.7 mm, as shown in Figure 3a. The valve core diameter is 4 mm and the total length is 9 mm. The valve core has four grooves with a thickness of 0.2 mm, a depth of 0.36 mm, and a total length of 7 mm, as shown in Figure 3b. The length of the groove is slightly less than the length of the baffle, and the depth of the groove is slightly higher than the maximum height of the baffle. The purpose is to prevent the groove and baffle from fully contacting and blocking the outlet. The inlet and outlet diameter of the connector are all 1 mm. The spring has a free length is 12 mm and is pre-stressed by an initial compression of 1 mm. The spring stiffness is 5 N/m.

### 2.2. Numerical Methods

In the flow analysis inside the MPV, the mass conservation equation and the momentum conservation equation are solved numerically by the software FLUENT, which can be written as

(2) $$\nabla \cdot \left(\rho \bar{v}\right)=0$$

(3) $$\frac{\partial }{\partial t}\left(\rho \bar{v}\right)+\nabla \cdot \left(\rho \bar{v}\bar{v}\right)=-\nabla p+\rho \left(\mu_{l}+\mu_{t}\right)\nabla^{2}\bar{v}+\rho \bar{g}$$

where $\;\bar{v}\;$ refers to the fluid velocity, *ρ* refers to the fluid density, $\mu_{l}$ and $\mu_{t}$ refer to molecular diffusivity (kinematic viscosity) and turbulent diffusivity, respectively, *p* refers to the fluid pressure, $\;\bar{g}\;$ refers to gravitational acceleration.

In this paper, the medium is liquid water with an incompressible and viscous. The Reynolds number is calculated by

(4) Re=ρv¯Dhμ≈197.66<2300

where *D_h_* refers to the hydraulic diameter. Thus, the fluid flow in the MPV is considered laminar, and the calculation is based on the laminar model.

**Figure 4:**
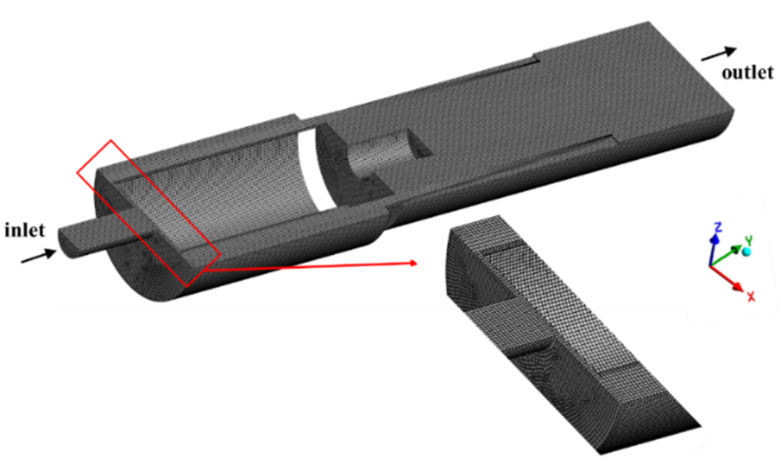
The mesh of flow domain.

**Figure 5:**
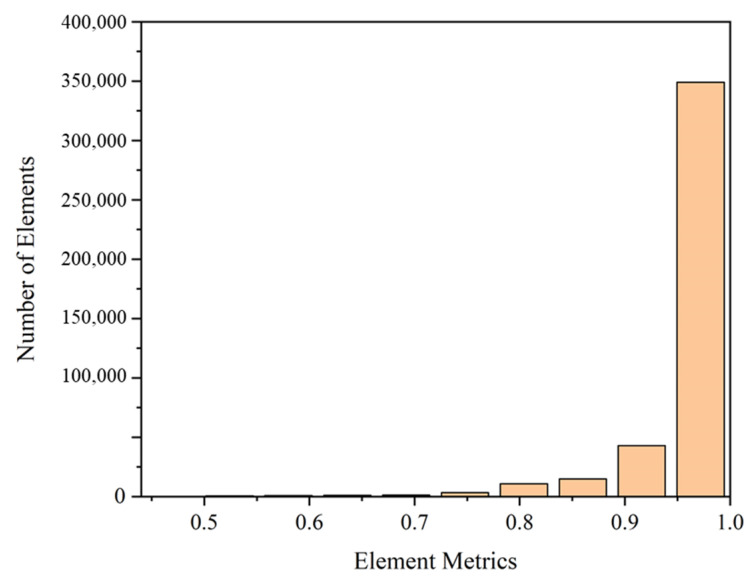
Quality check results of the generated mesh.

The flow domain is extracted from the MPV structure, with the hide of spring. Due to the symmetry of the flow domain, the 1/2 model is selected as the computational domain to improve calculation efficiency, as shown in Figure 4. The quality of the mesh is an important factor affecting the accuracy of numerical calculations. Mesh generation is performed through the ANSYS Workbench Mesh platform. The flow domain is discretized by the sweep method with hexahedral grids to reduce the number of grids and improve computational efficiency. The mesh size is 0.06 mm, and the number of mesh is 427,289. Figure 5 shows the quality check results of the generated mesh. The element quality is 82.1% close to 1 and the minimum value is 0.468. It shows that the reliability of the finite element analysis is guaranteed. In the numerical simulation of flow, the dynamic mesh method must keep the topology of its mesh unchanged, and at least one layer of the mesh must be retained in the valve gap at the moment of opening and closing of the valve. In this paper, the default opening is 0.1mm, and two layers of grids are reserved for the moment of opening.

The density and dynamic viscosity of liquid water are 998.2 kg/m^3^ and 0.00103 kg/(m·s), respectively. The gravity of the liquid water and valve core is ignored. The inlet boundary is defined as the pressure-inlet increasing linearly as a function of time, i.e., the inlet pressure ranges from 0 to 6.0 kPa with an increase of 0.5 kPa per second. The outlet pressure is set to atmospheric pressure. The dynamic mesh method and the User Defined Functions (UDF) are adopted. Friction between spool and body is ignored in UDF. Solving equations based on the SIMPLE algorithm are adopted.

To verify the reliability of numerical methods and simulation results, a comparison with similar simulations and experiments is conducted. Chappel et al. [33] conducted an experiment and simulation analysis of the flow rate of an MPV. The working principle and structure of the MPV in this paper are similar to those in the literature [33]. In the main parts of their paper, the inlet pressure with linearly increasing was adopted to simulate the flow characteristics in the valve, similarly, the same inlet boundary condition is chosen in this paper. Their research results show that the numerical results are in good agreement with the experimental results, and the flow rate of the valve remains constant over a range of pressures. Thus, conclusions obtained from this work can be seen as reliable.

## 3. Results and Discussion

### 3.1. Flow Rate of MPV

**Figure 6:**
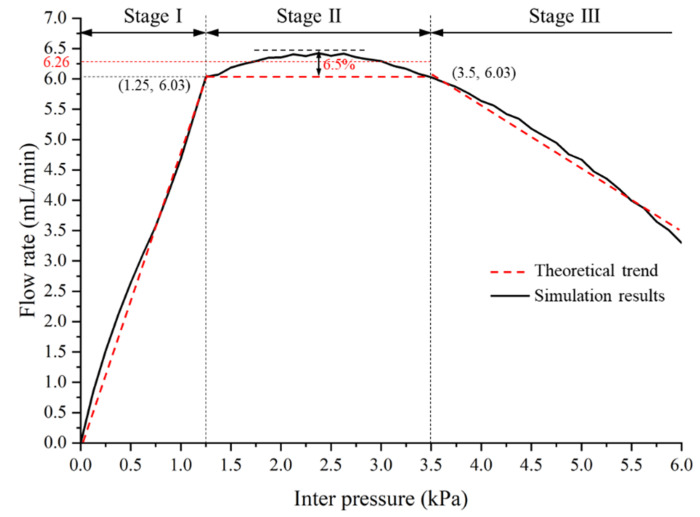
Flow rate of the MPV under varied inlet pressures.

**Figure 7:**
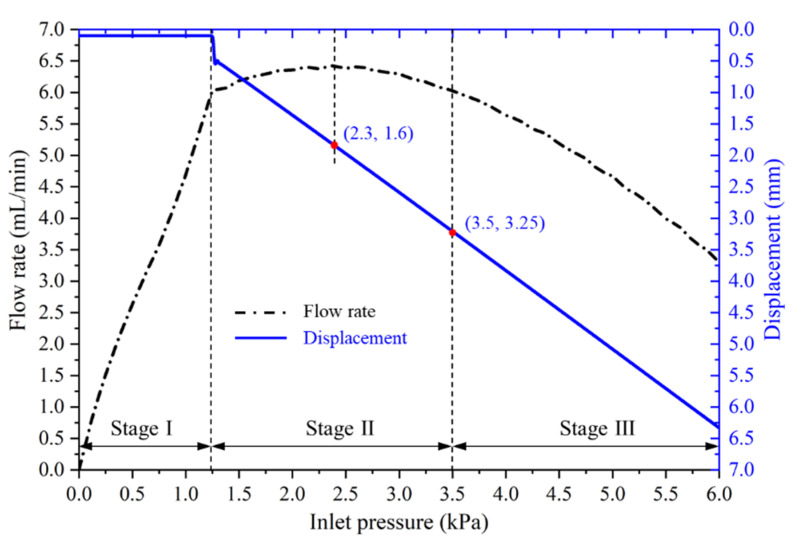
Displacement of the vale core under varied inlet pressures.

The flow rate of the MPV with varied inlet pressures is shown in Figure 6. It can be seen that the flow rate profile obviously has three-stage. For stage-Ⅰ, the flow rate increases almost linearly with the increase in inlet pressure. This is because the fluid force acting on the valve core is less than the preload force at △*P* < 1.25 kPa, and the valve core has not yet started to move, as shown in Figure 7. The MPV has a constant and low flow resistance in stage-Ⅰ. For the stage-Ⅱ, the flow rate is regulated at 6.03–6.42 mL/min for inlet pressure in the range from 1.25 kPa to 3.5 kPa. The error between the minimum and maximum flow rate is 6.5% and the average flow rate is 6.26 mL/min. It can be considered that a constant flow rate is maintained in the MPV in the stage-Ⅱ. The flow resistance linearly increases with the inlet pressure up to 3.5 kPa. For the stage-Ⅲ, the flow rate of the MPV gradually decreases with the increase in inlet pressure. This is because the gap distance between the groove and the baffle is gradually decreasing, and the resistance increases rapidly, i.e., the increment of flow resistance increment is significantly higher than the increment of inlet pressure, so the flow rate tends to decrease. The displacement of the valve core with varied inlet pressures is shown in Figure 7. It can be found that, when the inlet pressure reaches 3.5 kPa, the displacement of the valve core is 3.25 mm and the flow rate is 6.03 mL/min. As the displacement of the valve core increases, the flow resistance increases due to the reduction in the gap between the groove and the baffle. When the inlet pressure is 2.3 kPa, the maximum flow rate is 6.42 mL/min. At this time, the displacement of the valve core is 1.6 mm.

Therefore, in summary, when the pressure is 1.25–3.5 kPa, the constant flow rate maintains 6.26 mL/min. compared with the MPV that has been previously reported as shown in Table 1, the MPV in this work can achieve a higher flow rate at a much lower threshold pressure.

### 3.2. Flow Field Inside MPV

**Figure 8:**
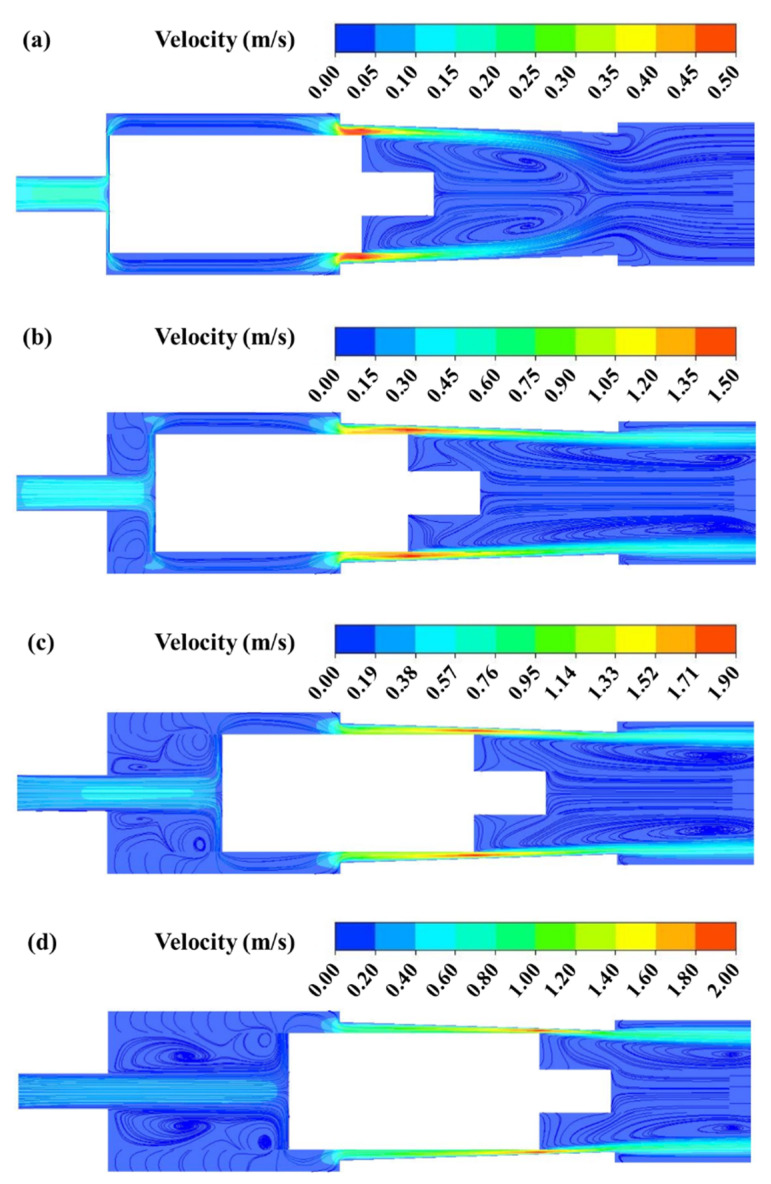
Velocity distribution under varied inlet pressures: (**a**) inlet pressure = 500 Pa; (**b**) inlet pressure = 2000 Pa; (**c**) inlet pressure = 3500 Pa; (**d**) inlet pressure = 5000 Pa.

In order to better study the flow characteristics of the MPV, the flow field in the valve is analyzed. The velocity distribution on the symmetry plane of the MPV with varied inlet pressures is shown in Figure 8. It can be seen that, after entering the MPV, the fluid flows to the inner storage cavity first. Next, the fluid separates into four streams and enters the outer storage cavity through the gap channel between the groove and the baffle. Four streams converge at the outer storage cavity and finally flow to the outlet of the MPV. When the fluid flows through the gap between the groove and the baffle, a jet is formed. The maximum velocity occurs at the gap channel between the groove and the baffle. With the increase in the inlet pressure, the maximum velocity gradually increases. However, the increment of the maximum velocity decreases with the increase in the inlet pressure. This is because the size of the gap channel between the groove and the baffle gradually decreases during the movement of the valve core, and flow resistance increases greatly. It also illustrates that the flow gradually decreases when the inlet pressure exceeds a certain pressure. When four-stream from the gap channel converge at the outer storage cavity, vortices appear near the bottom of the valve core. The vortices result from the jet flow from the gap channel. Meanwhile, there are also vortices in the inner storage cavity. Similarly, the vortices are also caused by the sudden expansion of the fluid domain.

**Figure 9:**
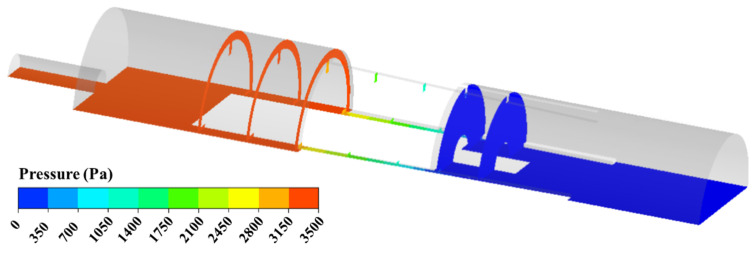
Pressure distribution with inlet pressure of 3.5 kPa.

**Figure 10:**
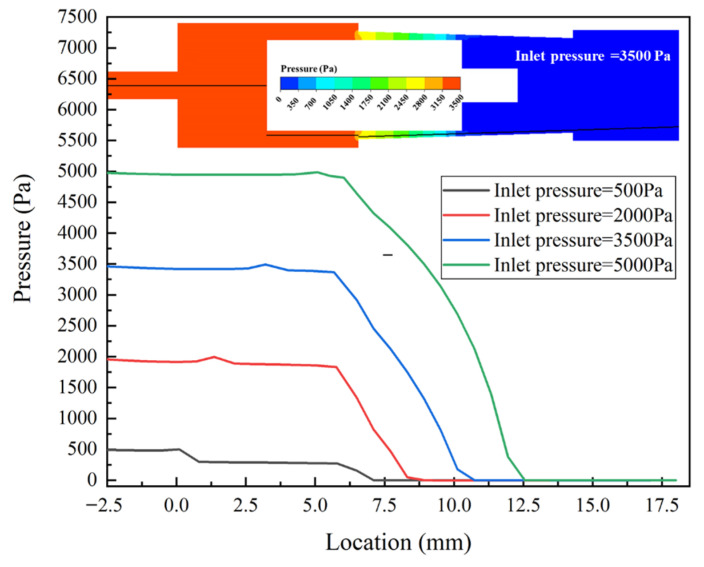
Pressure cures under varied inlet pressures.

The pressure distribution on the cross-section with the inlet pressure of 3.5 kPa is shown in Figure 9. It can be seen that there is a significant depressurization effect in the gap channel between the groove and baffle. The pressure of the inner and outer storage cavity is almost equal to the inlet pressure and outlet pressure, respectively. In order to obtain the pressure distribution of the MPV under varied inlet pressures, a straight line along the *y*-axis direction is established and the pressure data on it is extracted. Figure 10 shows the pressure distribution of the MPV with varied inlet pressures. It can be seen that, due to the difference in the position of the valve core, the pressure at the region of −2.5–6.5 mm is slightly different from the inlet pressure under different inlet pressures, especially the inlet pressure is 500 Pa. The pressure drop always mainly appears when *y* is in the range of 6.5–12.5 mm, i.e., the region is the gap channel between the groove and the baffle. With the movement of the valve core, the region of pressure drop becomes large.

## 4. Conclusions

In order to enable the MPV with high throughput liquid delivery, a novel structure of MPV with a constant flow rate independently of inlet pressure changes is proposed. The flow characteristics in the MPV are studied by numerical simulation. The difference between the fluid fore on the upper surface of the valve core and the spring force drives the valve core to move, thus adjusting the flow rate in the MPV. The constant flow of the MPV is maintained by automatically adjusting the size of the gap channel formed by the groove on the valve core and the baffle on the valve body. Under the inlet pressure varied from 1.25 kPa to 3.5 kPa, the constant flow rate maintained is 6.26 mL/min with a variation of 6.5%. Compared with the MPV that has been previously reported, the MPV in this work can achieve a higher flow rate at a much lower threshold pressure. With the increase in the inlet pressure, the maximum velocity gradually increases. However, the increment of the maximum velocity decreases with the increase in the inlet pressure. It also illustrates that the flow rate gradually decreases when the inlet pressure exceeds a certain pressure. The pressure drop always mainly appears in the gap channel between the groove and the baffle. With the movement of the valve core, the region of pressure drop becomes large.

## Figures and Tables

**Table 1:** The flow regulation capabilities of different MPVs.

**Type**	**Constant Flow Rate** **(mL/min)**	**Threshold Pressure** **(kPa)**
Push-up valve [39]	0.033	103
Check microfluidic valve [40]	1.20	100
Parallel membrane valve [41]	0.87	15
Passive flow control valve [33]	0.33	1
Low threshold pressure valve [42]	4.03	6
Microfluidic passive valve [43]	5.75	4
